# Do we need another prognostic score for cardiogenic shock patients with ECMO?

**DOI:** 10.1186/s13054-017-1753-7

**Published:** 2017-07-07

**Authors:** Sébastien Champion

**Affiliations:** Réanimation, Clinique de Parly 2, Ramsay Générale de Santé, 21 rue Moxouris, 78150 Le Chesnay, France

Chen et al. [1] succeeded in improving the SAVE score in patients who received extracorporeal membrane oxygenation (ECMO) for cardiogenic shock (CS) by simple addition of blood lactate. Accordingly, many other scores have been determined for outcome prediction for patients already receiving ECMO for CS; some reported by the authors and others being published afterward [2]. Enthusiasm in these scores is understandable but will not select adequate candidates for ECMO in the overall CS population. Chen et al*.* stated: “To avoid unnecessary use of ECMO, which might unnecessarily consume resources and expose patients to possible ECMO complications, thorough consideration must be used to identify the appropriate candidates for ECMO support” [1]. I strongly support and would like to emphasize their statement.

We designed a score based on cardiac power index (CPI, W/m^2^) and catecholamine level to predict death or use of ECMO in CS: this is the Catecholamine Refractoriness and Assistance guide based on cardiogenic Shock Hemodynamics (CRASH) score:

CRASH score = CPI/√[1 + Inotropic score (μg/kg/min) = dobutamine, dopamine + 100 × (noradrenaline + adrenaline) + 15 × (IPDE-3) + 10 for levosimendan

The CRASH score has a sensitivity of 68% and a specificity of 92% for death/ECMO. The area under the receiver operating characteristics curve was 0.851 with an overall accuracy of 0.833 with a 0.0375 threshold [3].

Our CRASH score is, in essence, a score of cardiac reserve that should have a role in defining refractory shock and in guiding mechanical circulatory support, provided hypoxia occurs. The addition of other elements, such as clinical (mottling, cyanosis, capillary refill time, rhythm, neurologic, respiratory, and hemodynamic variables and their kinetics) and biological (oxygen venous saturation, lactates, bicarbonates, platelets, prothrombin time, creatinine, interleukin-6, angiopoietins) data, and especially the etiology and etiological treatment of CS, may play a role in the prognostic assessment of patients. The ability of our CRASH score to quantify the severity of CS needs to be evaluated in large cohorts. Then, one could imagine a study evaluating the implementation of mechanical circulatory support (ECMO, Impella, or Tandemheart) according to two thresholds (a liberal threshold of 0.0375 or a restrictive threshold of 0.0300), or versus no assistance.

## Abbreviations

CPI: Cardiac power index
CRASH: Catecholamine Refractoriness and Assistance guide based on cardiogenic Shock Hemodynamics
CS: Cardiogenic shock
ECMO: Extracorporeal membrane oxygenation
